# Baseline total metabolic tumor volume combined with international peripheral T-cell lymphoma project may improve prognostic stratification for patients with peripheral T-cell lymphoma (PTCL)

**DOI:** 10.1186/s13550-020-00698-y

**Published:** 2020-09-23

**Authors:** Chong Jiang, Yue Teng, Jieyu Chen, Zhen Wang, Zhengyang Zhou, Chongyang Ding, Jingyan Xu

**Affiliations:** 1 Department of Nuclear Medicine, Nanjing Drum Tower Hospital, The Affiliated Hospital of Nanjing University Medical School, Nanjing, China; 2 Department of Pathology, Nanjing Drum Tower Hospital, The Affiliated Hospital of Nanjing University Medical School, Nanjing, China; 3 Department of Pathology, The First Affiliated Hospital of Nanjing Medical University, Jiangsu Province Hospital, Nanjing, China; 4 Department of Nuclear Medicine, The First Affiliated Hospital of Nanjing Medical University, Jiangsu Province Hospital, Nanjing, China; 5 Department of Hematology, Nanjing Drum Tower Hospital, The Affiliated Hospital of Nanjing University Medical School, Nanjing, China

**Keywords:** Peripheral T-cell lymphoma, Prognosis, Total metabolic tumor volume, PET/CT

## Abstract

**Purpose:**

The aim of this study was to explore the prognostic value of total metabolic tumor volume (TMTV) at baseline ^18^F-FDG PET/CT in patients diagnosed with peripheral T-cell lymphoma (PTCL).

**Materials and methods:**

Eighty-four newly diagnosed PTCL patients who underwent baseline ^18^F-FDG PET/CT prior to treatment between March 2009 and January 2019 were enrolled in this retrospective study. The FDG-avid lesions in each patient were segmented using semiautomated software to calculate the maximum standardized uptake value (SUVmax), total metabolic tumor volume (TMTV), and total lesion glycolysis (TLG) values using the boundaries of voxels presenting with the 41% SUVmax threshold method. Progression-free survival (PFS) and overall survival (OS) were used as end points to evaluate patient prognosis. The log-rank test and Cox regression analyses were used to evaluate PFS and OS.

**Results:**

ROC curve analysis indicated an ideal TMTV cut-off value of 228.8 cm^3^. During the 4–131 months (29.2 ± 28.5 months) follow-up period, high TMTV was significantly associated with worse PFS and OS. TMTV and the international peripheral T-cell lymphoma project score (IPTCLP) were independent predictors of PFS and OS with multivariate analysis. The combination of TMTV and the IPTCLP may provide significantly better risk substratification in PFS and OS of PTCL patients.

**Conclusions:**

Both TMTV and IPTCLP are independent predictors of PTCL patient survival outcomes. Moreover, the combination of TMTV and IPTCLP improved patient risk stratification and may contribute to personalized therapeutic regimens.

## Introduction

Peripheral T-cell lymphomas (PTCLs) constitute a heterogeneous and uncommon group of malignancies derived from post-thymic T cells or mature natural killer (NK) cells, representing approximately less than 15% of all non-Hodgkin lymphomas [1]. The nodal lymphoma group, as classified by the World Health Organization, contains four subtypes: peripheral T-cell lymphoma (PTCL) not otherwise specified (PTCL-NOS), angioimmunoblastic T-cell lymphoma (AITL) and anaplastic large-cell lymphoma (ALCL), either ALK-positive or ALK-negative [2]. The treatment outcome following cyclophosphamide, doxorubicin, vincristine and prednisolone (CHOP) or CHOP-like regimens as first-line chemotherapy in patients with peripheral T-cell lymphoma (PTCL) is still unsatisfactory and is associated with a high failure rate and frequent relapses [3]. Therefore, an accurate prognostic method is urgently needed to risk-stratify patients and to tailor therapies to individual patients.

The prognostic index for PTCL (PIT), including 4 clinical characteristics (age, Eastern Cooperative Oncology Group performance status [ECOG PS], serum lactate dehydrogenase [LDH] and bone marrow [BM] involvement) as a basic tool for risk stratification of PTCL, has been widely used in clinical practice [4]. However, a new score taking age, performance status, and platelet count into consideration as main variables introduced by Vose was presented as the International peripheral T cell lymphoma Project (IPTCLP) in PTCL patients, but the prognostic value has been verified by only a few studies [5–7].

New volume-based parameters derived from baseline ^18^F-FDG PET/CT, such as total metabolic tumor volume (TMTV) and total lesion glycolysis (TLG), can reflect metabolic volume and activity and have been proposed as quantitative indexes of tumor metabolism biomarkers in Hodgkin’s and B-cell lymphomas [8]. However, PET data in peripheral T-cell lymphoma (PTCL) are still limited. Therefore, the aim of the current study was to explore the prognostic value of quantitative indexes derived from baseline PET/CT in newly diagnosed PTCL patients.

## Materials and methods

### Patients

Patients recently diagnosed with PTCL (including PTCL-NOS, AITL, ALCL ALK negative) who underwent pretreatment whole-body ^18^F-FDG PET/CT between March 2009 and January 2019 were enrolled in this retrospective study. To be included, patients were required to be treated with CHOP, a CHOP-like regimen or dose-adjusted etoposide, prednisone, vincristine, cyclophosphamide and doxorubicin (DA-EPOCH) regimens with a curative intent and had to be free of any concurrent diseases that precluded the protocol treatment. Patients were excluded if they had a previous malignancy, chemotherapy, radiotherapy, pregnancy (lactation), or diabetes mellitus with a fasting blood glucose level greater than 150 mg/dL. In addition, ALCL patients with ALK positivity who had a different treatment (ALK inhibitor therapy), and those with relatively favorable prognosis were excluded. Clinical parameters (sex, age, B symptoms, ECOG PS, Ann Arbor Stage, LDH level, platelet count, bone marrow biopsy [BMB] results and Ki-67) were determined from the medical records. Approval was obtained from the Ethics Committee of Nanjing Drum Tower Hospital, the Affiliated Hospital of Nanjing University Medical School. All of the subjects signed a written consent form.

### Three prognostic scores in PTCL

According to the criterion previously described [4, 5, 9], the three score systems (IPI, PIT and IPTCLP) were calculated. The IPI includes five variables: age (≤ 60 vs > 60), performance status ECOG (≤ 1 vs > 2), LDH level (low vs high), Ann Arbor stage (I–II vs III–IV) and extranodal involvement (≤ 1 vs > 2). Four risk groups were defined by IPI: score 0–1, low risk; score 2, low-intermediate risk; score 3, high-intermediate risk and score 4–5, high risk, respectively. The PIT includes 4 variables: age (≤ 60 vs > 60), performance status (ECOG ≤ 1 vs > 2), LDH level (low vs high) and BM involvement (negative versus positive). Four risk groups were defined by PIT: score 0, low risk; score 1, low-intermediate risk; score 2, high-intermediate risk and score ≥ 3, high risk, respectively. The IPTCLP includes 3 variables: age (≤ 60 vs > 60), ECOG performance status (ECOG PS ≤ 1 vs > 2) and platelet cell count (< 150 × 10^9^/l vs ≥ 150 × 10^9^/L). Four risk groups were defined by IPTCLP: score 0, low risk; score 1, low-intermediate risk; score 2, high-intermediate risk and score 3, high risk, respectively. For the purpose of this study, the four risk groups were dichotomized into low-risk IPI, PIT and IPTCLP (comprising low- and low-intermediate-risk patients) and high-risk (comprising high-intermediate- and high-risk patients) groups.

### PET/CT scanning protocol

All of the patients underwent whole-body ^18^F-FDG PET/CT on a combined Gemini GXL PET/CT scanner with a 16-slice CT component (Philips Corp, Netherlands). After 6 h of fasting (no oral or intravenous fluids containing sugar or dextrose), 185–370 MBq of ^18^F-FDG (5.18 MBq/kg) was administered intravenously. Each patient’s blood glucose level was checked immediately before ^18^F-FDG administration. Each patient was weighed for determination of the standardized uptake value (SUV) prior to each scan. Whole-body PET/CT scans (from the base of the skull to the upper thigh) were started 60 min following radiopharmaceutical injection. Emission data were acquired for 2 min in each bed position. CT acquisition data were used for attenuation correction, and corrected PET images were reconstructed using ordered-subset expectation maximization (OSEM). The matrix size is 144 × 144. The acquired images from the PET and CT scans were sent for image registration and fusion using Syntegra software.

### Imaging analysis

PET/CT images were read by two physicians specializing in nuclear medicine. These physicians were blind to any patient information or to any of the patient's clinical conditions. When in doubt, the results were determined by a consensus between the two physicians. Images were reviewed using volume-viewer software on a dedicated workstation (Compassview 5.0, Philips Corp, the Netherlands) to calculate SUV and MTV. Regions of interest (ROIs) were placed manually to cover the lesion, and the maximum SUV (SUVmax) value was recorded for each lesion. For each PET dataset, the SUVmax was defined as the highest SUV among all of the hypermetabolic tumor foci. MTV was determined by drawing a circular ROI fully encasing all involved lesions in axial, coronal, and sagittal PET/CT images. Then, the boundaries of voxels were produced automatically with the 41% SUVmax threshold method recommended by the European Association of Nuclear Medicine [10]. Normal organs and false-positive lesions—such as inflammation, infection or other benign FDG-avid lesions based on histopathological reports or other imaging modalities—were subtracted. The TMTV was obtained by summing the MTV of all lesions. TLG was calculated as the sum of all MTV × SUV (mean of lesions) in each patient. SUVmax values were obtained and corrected for body weight using the following standard formula: mean ROI activity (MBq/mL)/[injected dose (MBq)/body weight (kg)].

### Statistical methods

Progression-free survival (PFS) and overall survival (OS) were chosen as the end points to evaluate the prognoses of PTCL patients. PFS was defined as the interval between the date of diagnosis and the dates of first relapse, progression, death from any cause, or last follow-up. OS was defined as the interval from the date of diagnosis until the time of death from any cause or last follow-up. Receiver operating characteristic (ROC) curves were constructed to estimate the accuracies in predicting ideal cut-off values for SUVmax, TMTV and TLG. Estimations of sensitivity and specificity were based on these cut-off values. Characteristics of the population were compared between groups using Pearson’s chi-square test. The distributions of PFS and OS rates were estimated using the Kaplan–Meier method, and the survival curves were compared by a log-rank test. For the significant PET and clinical variables in univariate analysis, multivariate analysis using the Cox proportional hazards model was performed to assess the potential independent effects on PFS and OS. All of the statistical analyses were performed using SPSS 22.0, and a *P* value less than 0.05 was considered to be statistically significant.

## Results

### Patient characteristics and treatment results

Eighty-four patients (30 women and 54 men), including 47 patients with PTCL-NOS, 30 with AITL, and 7 with ALCL (ALK negative), were included in this study. Their clinical characteristics are summarized in Tables 1 and 2. The median patient age was 62 years (range 16–85 years). The average SUVmax, TMTV and TLG of the primary tumors were 11.2 (2.6–33.5), 347.3 (3.4–1687.0) cm^3^ and 1043.3 (10.9–6308.0), respectively. After a median follow-up of 20.0 months (range 4–131 months), 47 patients had disease relapse or progression, and 46 patients died.

**Table 1:** Demographics and clinical characteristics of the study population

Characteristics	Overall patients, *n* = 84
Sex	
Female/male	30/54
Age	
≤ 60 years/> 60 years	43/41
LDH	
Normal/higher than normal	38/46
B symptoms	
No/yes	33/51
ECOG performance status	
0–1/> 1	57/27
Ann arbor stage	
I–II/III–IV	15/69
Extranodal sites ≥ 2	
No/yes	65/19
BMB	
Negative/positive	63/21
Ki-67 ≥ 80%	
No/yes	60/24
Platelet cell count ≥ 150 × 10^9^/L	
No/yes	48/36
IPI	
0–2/3–5	47/37
PIT	
0–1/2–4	41/43
IPTCLP	
0–1/2, 3	54/30

*LDH* lactate dehydrogenase, *ECOG PS* Eastern Cooperative Oncology Group performance status, *BMB* bone marrow biopsy, *IPI* International Prognostic Index, *PIT* prognostic index for T-cell lymphoma, *IPTCLP* International peripheral T cell lymphoma Project

**Table 2:** Clinical and PET characteristics of different PTCL subtypes

Characteristics	PTCL-NOS	AITL	ALCL (ALK^−^)
Sex: female/male	18/29	9/21	3/4
Age: ≤ 60 years/> 60 years	25/22	12/18	6/1
LDH: normal/higher than normal	26/21	9/21	3/4
B symptoms: no/yes	21/26	8/22	4/3
ECOG performance status: 0–1/> 1	29/18	21/9	7/0
Ann Arbor stage: I–II/III–IV	10/37	2/28	3/4
Extranodal sites ≥ 2: no/yes	33/14	25/5	7/0
BMB: negative/positive	35/12	22/8	6/1
Ki-67 ≥ 80%: no/yes	35/12	22/8	6/1
Platelet cell count ≥ 150 × 10^9^/L: no/yes	24/23	22/8	2/5
SUVmax	10.6 (2.6–25.5)	11.3 (3.9–25.5)	14.3 (4.3–33.5)
TMTV (cm^3^)	277.7 (3.4–1334.6)	521.5 (4.3–1887.0)	67.8 (3.8–250.0)
TLG	638.1 (10.9–3255.9)	1825.5 (10.9–6308.0)	411.1 (14.9–1182.9)

*PTCL-NOS* peripheral T-cell lymphoma (PTCL) not otherwise specified, *AITL *angioimmunoblastic T-cell lymphoma, *ALCL* anaplastic large-cell lymphoma, *LDH* lactate dehydrogenase, *ECOG PS* Eastern Cooperative Oncology Group performance status, *BMB* bone marrow biopsy

### ROC curve analysis of SUVmax, TMTV and TLG

In the present study, ROC curve analysis was used to calculate the accuracy of ideal cut-off values to distinguish a low SUVmax group from a high SUVmax group, a low TMTV group from a high TMTV group and a low TLG group from a high TLG group. The estimated area under the ROC curve (AUROC) for SUVmax was 0.617, that for TMTV was 0.797 and that for TLG was 0.696. The ideal cut-off values for SUVmax, TMTV and TLG were 6.9, 228.8 cm^3^ and 437.3, respectively. The sensitivity, specificity, accuracy, positive predictive value and negative predictive value in predicting PFS and OS are listed in Table 3.

**Table 3:** Prediction of outcomes with SUVmax, TMTV and TLG

	Progression-free survival			Overall survival		
	SUVmax	TMTV	TLG	SUVmax	TMTV	TLG
Se (%)	83.0	72.3	74.5	83.0	73.9	76.1
Sp (%)	37.8	81.1	62.2	39.5	81.6	63.2
Acc (%)	63.1	76.2	69.0	64.3	77.4	70.2
PPV (%)	62.9	82.9	71.4	62.9	82.9	71.4
NPV (%)	63.6	69.8	65.7	65.2	72.1	68.6

*Se* sensitivity, *Sp* specificity, *Acc* accuracy, *PPV* positive predictive value, *NPV* negative predictive value, *SUVmax* maximum standardized uptake value, *TMTV* total metabolic tumor volume, *TLG* total lesion glycolysis

### Clinical characteristics of patients in relation to TMTV and TLG

Table 4 shows the differences in clinical characteristics between the dichotomized TMTV and TLG groups. Patients with high TMTV and TLG usually possessed the following characteristics: high IPI and PIT scores. In addition, the results showed that the B symptoms and ECOG PS were significantly associated with TMTV, and LDH level was significantly associated with TLG.

**Table 4:** Comparison of patient clinical data with TMTV and TLG

Variable	No. of patients (*n* = 84)	TMTV			TLG		
		Low (*n* = 43)	High (*n* = 41)	*P* value*	Low (*n* = 35)	High (*n* = 49)	*P* value*
Sex, F/M	30/54	15/28	15/26	1.000	14/21	16/33	0.499
Age, ≤ 60/> 60	43/41	25/18	18/23	0.275	21/14	22/27	0.191
LDH level, normal/elevated	38/46	24/19	14/27	0.052	21/14	17/32	0.027
B symptoms, no/yes	33/51	23/20	10/31	0.008	18/17	15/34	0.071
ECOG PS, 0–1/ ≥ 2	57/27	35/8	22/19	0.010	28/7	29/20	0.059
Ann Arbor stage, I–II/III–IV	15/69	11/32	4/37	0.087	10/25	5/44	0.043
Extranodal sites ≥ 2, no/yes	65/19	36/7	29/12	0.196	29/6	36/13	0.429
BMB, negative/positive	63/21	36/7	27/14	0.079	28/7	35/14	0.449
Ki-67 ≥ 80%, no/yes	60/24	33/10	27/14	0.336	26/9	34/15	0.807
Platelet cell count, ≥ 150 × 10^9^/L	48/36	24/19	24/17	0.829	22/13	26/23	0.503
IPI, 0–2/3–5	47/37	34/9	13/28	< 0.001	27/8	20/29	0.002
PIT, 0–1/2–4	41/43	29/14	12/29	0.001	24/11	17/32	0.004
IPTCLP, 0–1/2, 3	54/30	31/12	23/18	0.172	26/9	28/21	0.165

A chi-square test was used to test the significance of the association between clinical data and the baseline TMTV and TLG

*LDH* lactate dehydrogenase, *ECOG PS* Eastern Cooperative Oncology Group performance status, *BMB* bone marrow biopsy, *IPI* International Prognostic Index, *PIT* prognostic index for T-cell lymphoma, *IPTCLP* International peripheral T cell lymphoma Project, *MTV* metabolic tumor volume, *TLG* total lesion glycolysis

*P < 0.05

### Survival analysis for FDG PET/CT metrics and the IPTCLP scores

**Fig. 1:**
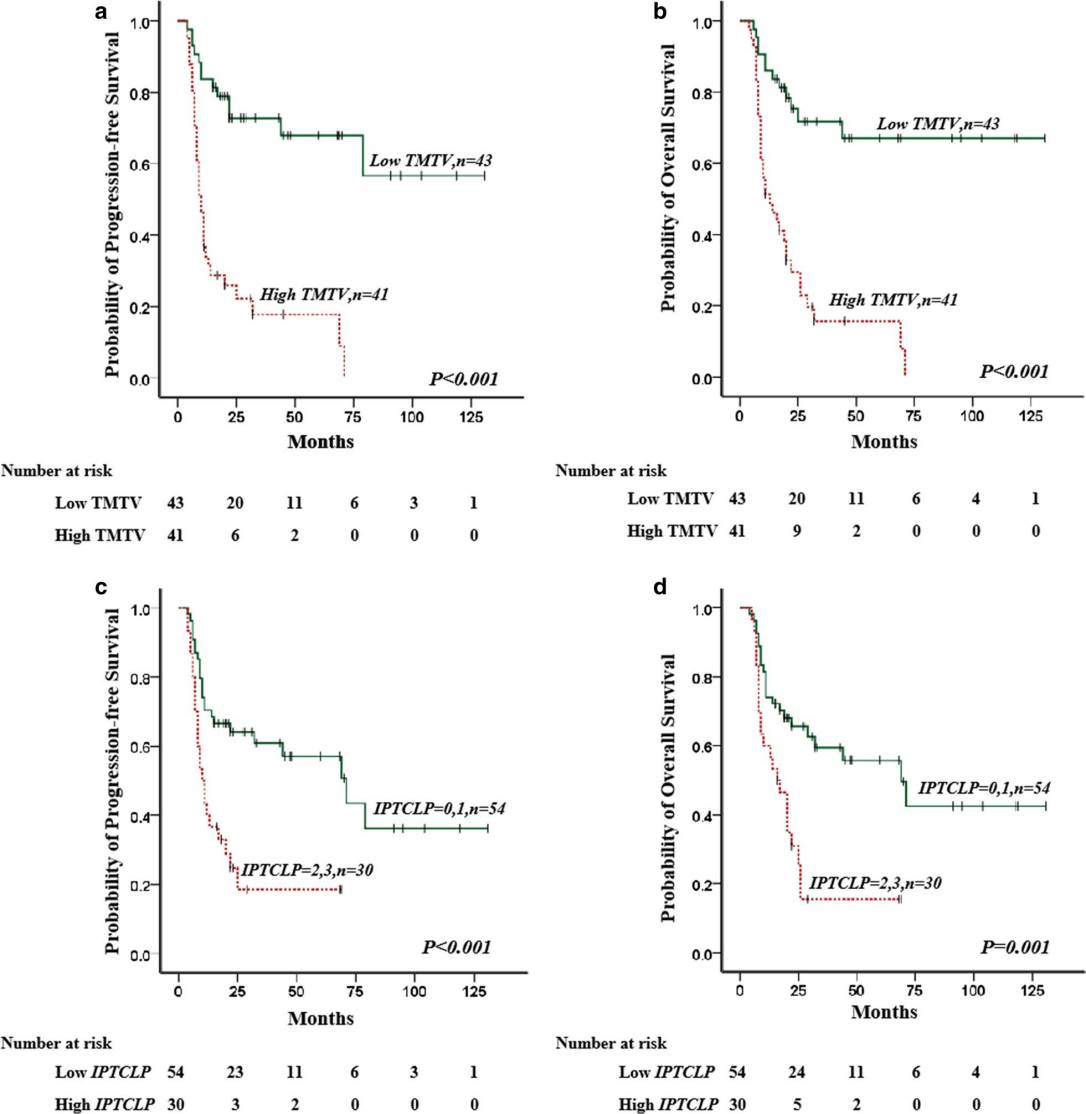
Overall survival (OS) and progression-free survival (PFS) of patients with peripheral T-cell lymphoma. **a**, **b** The PFS and OS of the PTCL patients could be successfully distinguished by TMTV (*P* < 0.001 and *P* < 0.001). **c**, **d** The PFS and OS of the PTCL patients could be successfully distinguished by IPTCLP scores (*P* < 0.001 and *P* = 0.001)

The mean PFS was 55.0 mo (95% CI: 41.1–68.9 months, range: 4–79 months), and the mean OS was 57.4 months (95% CI 43.5–71.2 months, range 4–71 months). The PFS and OS estimates for all of the patients were 44.0% and 45.2%, respectively. Univariate analysis showed that B symptoms, BMB positive result, a high LDH level, high ECOG PS, high platelet cell count, high IPI score, high PIT score, high IPTCLP score, high SUVmax, high TMTV and high TLG were significantly correlated with inferior PFS and OS. The survival curves and univariate analyses are shown in Fig. 1 and Table 5. TMTV and IPTCLP were independent predictors of both PFS [HR (95% CI): 5.096 (2.579–10.072), *P* < 0.001; HR (95% CI): 2.577 (1.405–4.727), *P* = 0.002] and OS [HR (95% CI): 4.647 (2.361–9.148), *P* < 0.001; HR (95% CI): 2.360 (1.285–4.336), *P* = 0.006] after multivariate analysis (Table 6).

**Table 5:** Univariate analysis of factors predictive of progression-free and overall survival

Variable	No. of patients (*n* = 84)	Progression-free survival					Overall survival				
		*B*	SE	Wald	HR (95% CI)	*P* value*	*B*	SE	Wald	HR (95% CI)	*P* value*
Sex, F/M	30/54	0.334	0.315	1.124	1.396 (0.753–2.588)	0.289	0.374	0.323	1.342	1.454 (0.772–2.738)	0.247
Age, ≤ 60/> 60	43/41	0.513	0.304	2.841	1.670 (0.920–3.032)	0.092	0.576	0.305	3.564	1.779 (0.978–3.236)	0.059
LDH level, normal/elevated	38/46	0.679	0.310	4.790	1.972 (1.073–3.621)	0.029	0.800	0.317	6.389	2.226 (1.197–4.141)	0.011
B symptoms, no/yes	33/51	0.861	0.334	6.662	2.366 (1.230–4.550)	0.010	0.840	0.341	6.072	2.317 (1.188–4.520)	0.014
ECOG PS, 0–1/≥ 2	57/27	1.055	0.300	12.337	2.873 (1.594–5.176)	< 0.001	0.999	0.301	10.986	2.715 (1.504–4.900)	0.001
Ann Arbor stage, I–II/III–IV	15/69	0.867	0.477	3.300	2.379 (0.934–6.059)	0.069	0.975	0.526	3.436	2.650 (0.946–7.429)	0.064
Extranodal sites ≥ 2, no/yes	65/19	0.545	0.321	2.880	1.724 (0.919–3.233)	0.090	0.494	0321	2.369	1.639 (0.874–3.075)	0.124
BMB, negative/positive	63/21	0.652	0.310	4.430	1.920 (1.046–3.525)	0.035	0.624	0.310	4.050	1.866 (1.016–3.427)	0.044
Ki-67 ≥ 80%, no/yes	60/24	0.556	0.305	3.334	1.744 (0.960–3.169)	0.068	0.517	0.306	2.854	1.678 (0.920–3.057)	0.091
Platelet cell count, ≥ 150 × 10^9^/L	48/36	0.594	0.300	3.923	1.812 (1.006–3.262)	0.048	0.588	0.300	3.836	1.800 (1.000–3.240)	0.050
IPI, 0–2/3–5	47/37	1.148	0.313	13.475	3.153 (1.708–5.820)	< 0.001	1.143	0.314	13.287	3.136 (1.696–5.799)	< 0.001
PIT, 0–1/2–4	41/43	1.068	0.317	11.316	2.909 (1.561–5.418)	0.001	1.113	0.324	11.799	3.044 (1.613–5.744)	0.001
IPTCLP, 0–1/2, 3	54/30	1.027	0.304	11.445	2.793 (1.540–5.064)	0.001	0.999	0.304	10.763	2.715 (1.495–4.930)	0.001
SUVmax, low/high	22/62	0.959	0.405	5.619	2.609 (1.181–5.766)	0.018	1.020	0.421	5.878	2.773 (1.216–6.325)	0.015
TMTV, low/high	43/41	1.665	0.344	23.460	5.286 (2.695–10.369)	< 0.001	1.608	0.342	22.095	4.994 (2.554–9.766)	< 0.001
TLG, low/high	35/49	1.201	0.351	11.680	3.324 (1.669–6.620)	0.001	1.179	0.351	11.273	3.250 (1.633–6.467)	0.001

Univariate analyses of factors predictive of survival in patients whose scans were evaluated using TMTV and TLG

*CI* confidence interval, *SE* standard error, *LDH* lactate dehydrogenase, *ECOG PS* Eastern Cooperative Oncology Group performance status, *BMB* bone marrow biopsy, *IPI* International Prognostic Index, *PIT* prognostic index for T-cell lymphoma, *IPTCLP* International peripheral T cell lymphoma Project, *TMTV* total metabolic tumor volume, *TLG* total lesion glycolysis

*P < 0.05

**Table 6:** Multivariate analysis of predictors of progression-free and overall survival

Variable	Progression-free survival					Overall survival				
	*B*	SE	Wald	HR (95% CI)	*P *value*	*B*	SE	Wald	HR (95% CI)	*P* value*
LDH level, normal/elevate	–	–	–	–	0.472	–	–	–	–	0.233
B symptoms, no/yes	–	–	–	–	0.233	–	–	–	–	0.469
ECOG PS, 0–1/ ≥ 2	–	–	–	–	0.708	–	–	–	–	0.988
BMB, negative/positive	–	–	–	–	0.932	–	–	–	–	0.999
Platelet cell count, ≥ 150 × 10^9^/L	–	–	–	–	0.276	–	–	–	–	0.289
IPI, 0–2/3–5	–	–	–	–	0.888	–	–	–	–	0.827
PIT, 0–1/2–4	–	–	–	–	0.825	–	–	–	–	0.632
IPTCLP, 0–1/2, 3	0.947	0.310	9.351	2.577 (1.405–4.727)	0.002	0.859	0.310	7.664	2.360 (1.285–4.336)	0.006
SUVmax, low/high	–	–	–	–	0.689	–	–	–	–	0.688
TMTV, low/high	1.629	0.348	21.953	5.096 (2.579–10.072)	< 0.001	1.536	0.346	19.766	4.647 (2.361–9.148)	< 0.001
TLG, low/high	–	–	–	–	0.964	–	–	–	–	0.933

Univariate analyses of factors predictive of survival in patients whose scans were evaluated using TMTV and TLG

*CI* confidence interval, *SE* standard error, *CI* confidence interval, *HR* hazards ratio, *LDH* lactate dehydrogenase, *ECOG PS* Eastern Cooperative Oncology Group performance status, *BMB* bone marrow biopsy, *IPI* International Prognostic Index, *PIT* prognostic index for T-cell lymphoma, *IPTCLP* International peripheral T cell lymphoma Project, *TMTV* total metabolic tumor volume, *TLG* total lesion glycolysis

*P < 0.05

### Survival analysis for the combination of TMTV and the IPTCLP scores

**Fig. 2:**
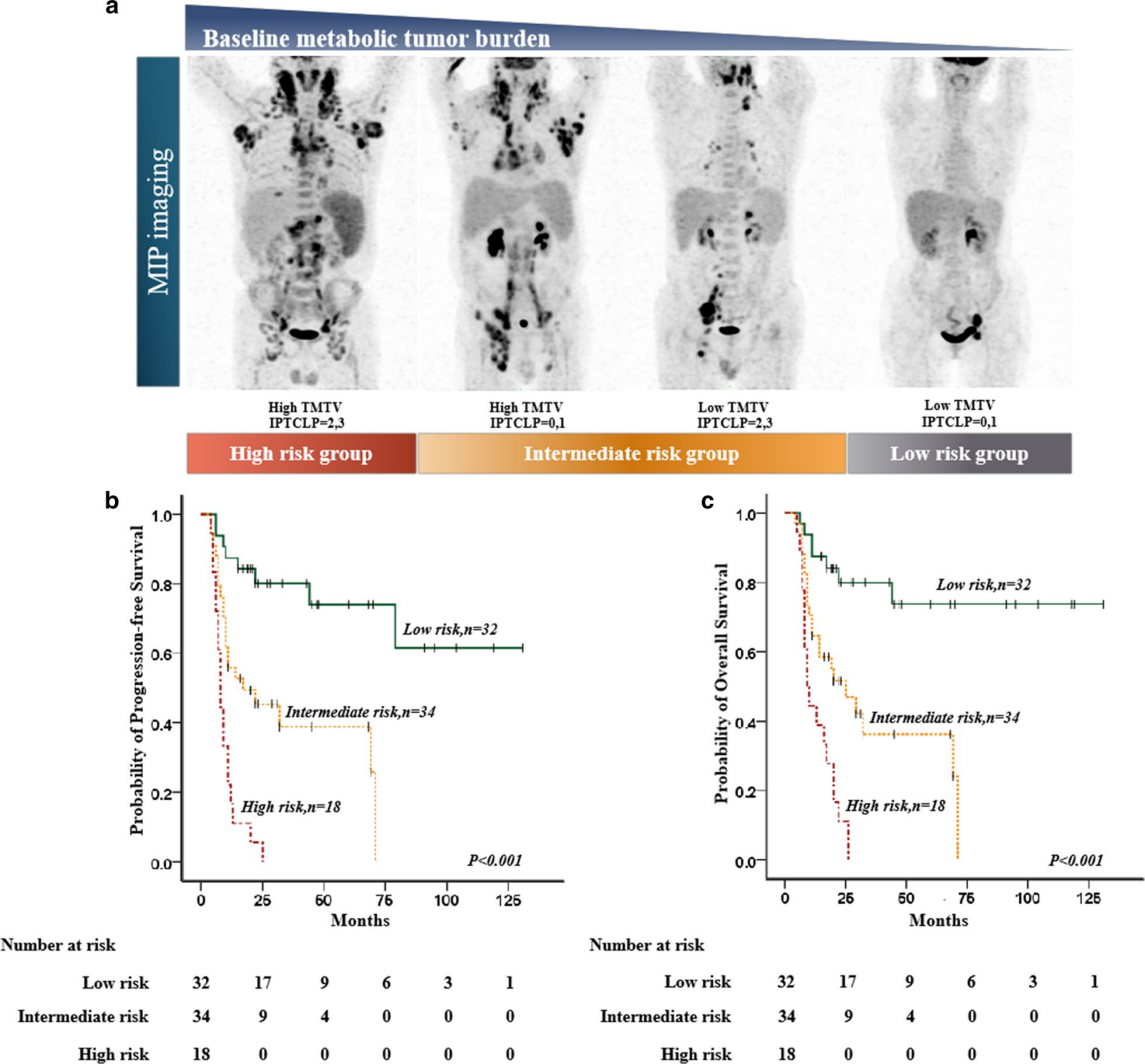
**a** Illustration of combination of total metabolic tumor volume and IPTCLP scores for risk substratification of PTCL patients using maximal intensity projection on FDG-PET images. **b**, **c** Three risk groups with the combination of baseline TMTV and IPTCLP scores: low-risk group (IPTCLP = 0, 1 and low TMTV, *n* = 32), intermediate-risk group (IPTCLP = 2, 3 or high TMTV, *n* = 34), and high-risk group (IPTCLP = 2, 3 and high TMTV, *n* = 18)

The baseline TMTV was added to the IPTCLP score systems, and patients were divided into three risk groups with significantly different PFS (χ^2^ = 39.795, *P* < 0.001) and OS (*χ*^2^ = 35.871, *P* < 0.001) values. (Fig. 2). In a subanalysis, the high-risk group (TMTV > 228.8 cm^3^ and IPTCLP score of 2, 3) had relatively lower survival than those in the low-risk group (TMTV ≤ 228.8 cm^3^ and IPTCLP score of 0–1) and intermediate-risk group (TMTV > 228.8 cm^3^ or IPTCLP score of 2, 3) (PFS: *χ*^2^ = 42.120, *P* < 0.001; *χ*^2^ = 13.322, *P* < 0.001; OS: *χ*^2^ = 36.056, *P* < 0.001; *χ*^2^ = 10.883, *P* = 0.001). In addition, the intermediate-risk group (TMTV > 228.8 cm^3^ and IPTCLP score of 2, 3) also had relatively lower survival than those in the low-risk group (TMTV ≤ 228.8 cm^3^ and IPTCLP score of 0–1) (PFS: *χ*^2^ = 12.512, *P* < 0.001; OS: *χ*^2^ = 12.262, *P* < 0.001). Outcomes according to the combination of TMTV and the IPTCLP are listed in Table 7.

**Table 7:** Risk stratification and outcomes of progression-free and overall survival

Risk group	Risk factors	Patients	Events	Progression-free survival (%)	Events	Overall survival (%)
TMTV combined with IPTCLP						
Low risk	Low TMTV and IPTCLP = 0, 1	32/84	8	75.0	7	78.1
Intermediate risk	High TMTV or IPTCLP = 2, 3	34/84	21	38.2	21	38.2
High risk	High TMTV and IPTCLP = 2, 3	18/84	18	0	18	0

## Discussion

Our results confirm the strong prognostic value of baseline TMTV in patients with PTCL, and patients with a TMTV greater than 228.8 cm^3^ had lower survival. This result is consistent with the results of published studies [11, 12]. In the study of Cottereau et al., the baseline TMTV (cutoff value of 230 cm^3^) was found to be the only significant independent predictor for both PFS (*P* = 0.013) and OS (*P* = 0.021) [11]. Mehta-Shah et al.’s study also showed that a high baseline TMTV (cutoff value is 125 cm^3^) predicted worse OS (HR 6.025; *P* = 0.022) and EFS (HR 3.861; *P* = 0.005) [12]. TMTV is a measure of the viable tumor fraction and may better represent the metabolic burden of tumors. The discrepancy between the optimal thresholds in Mehta-Shah et al.’s study compared to those in our present study can be explained by the different therapy regimens, as patients in their studies received CHOP or CHOEP regimen with autologous transplant as consolidation. SUVmax is the most commonly used semiquantitative index of ^18^F-FDG uptake, reflecting the tumor glucose metabolism of the most aggressive cell component, and previous studies have suggested an association between SUVmax and tumor aggressiveness [13, 14]. However, SUVmax was not found to be associated with outcome in our study, probably because FDG avidity at baseline is variable in patients with PTCL [15, 16].

Initially designed for risk stratification in aggressive lymphomas, the IPI is the most commonly used prognostic score system for patients with aggressive PTCL [17]. However, the usefulness of the IPI in PTCL has been questioned in some studies [18, 19]. To better define the clinical outcome, several prognostic score systems, including the PIT and IPTCLP, were built for PTCL patients. The predictive capacity of the PIT score has been verified in PTCL-NOS in a manner similar to that seen in diffuse large B-cell lymphoma [4]. More recently, the IPTCL was developed and reported to have a better performance than PIT scores to predict the outcome of PTCL patients in Garcĺa et al.’s study [6]. Although all three scores demonstrated their ability to predict the outcome of patients with PTCL in our study, no dramatic differences were observed among the indexes in our study, and the IPTCL was shown to be better than the other two scores to predict survival outcomes in the multivariate analysis.

The treatment outcomes of patients with PTCL were worse than those with aggressive B-cell lymphomas, with early relapse, PFS of less than 1 year, and OS of less than 2 years [15, 20]. Moreover, a small proportion of patients who can survive for long periods of time or even be cured was also reported [21, 22]. Therefore, an accurate prognostic assessment is urgently needed for PTCL patients to better select high-risk patients as well as potentially curable patients. Some studies have reported that pretreatment PET/CT parameters can give added prognostic value to prognostic score systems to better stratify the progression risk of lymphoma patients [23, 24]. Cottereau et al. found that the addition of TMTV to PIT could identify different risk categories of PTCL patients [11]. In the present study, we added a baseline TMTV into the IPTCLP to stratify patients into three distinct prognostic groups. This resulted in the identification of three groups of patients with significantly different outcomes. This study demonstrated that baseline TMTV could be used for further precise prediction of PTCL patient prognosis when combined with IPTCLP scores.

The results among studies might be inconsistent due to the different thresholds used for delineating tumors. In some studies, the absolute threshold of an SUV ≥ 3.0 or 2.5 was used to calculate MTV [12, 25, 26] and proved to be easiest to apply in clinical settings [27]. In addition, PTCL is heterogeneous in their FDG-uptake, and TMTV estimated using fixed value thresholding (SUV ≥ 3.0 or 2.5) may reflect the total tumor burden more accurately. However, we calculated MTV using an 41% SUVmax as the ROI absolute threshold, as in previous studies [28, 29]. Actually, the 41% SUVmax threshold method has been recommended by the European Association of Nuclear Medicine due to the better interobserver agreement [10]. However, a consensus on the MTV calculation method is still lacking, and an accurate and normalized method for defining metabolic volume is needed in the future [30].

This study was constrained by its retrospective nature. Because of the limited number of patients in the present study, we considered patients with PTCL as a whole, and the histological subtypes were not further evaluated. Although sharing a common T-cell origin and aggressive behavior with poor outcome, subtypes have a particular clinico-biological personality. In addition, various first-line treatments used in the patients may cause bias that confounded the analysis of our results. Therefore, a prospective clinical trial with a larger sample size of PTCL patients is needed to provide a more reliable prediction of survival in such patients.

## Conclusion

Both TMTV and IPTCL are independent predictors of the PTCL patient survival outcome. Moreover, the combination of TMTV and IPTCLP scores improved patient risk stratification and might contribute to the ability to personalize therapeutic regimens.

## Data Availability

The datasets generated and analyzed during the current study are available in the Nanjing Drum Tower Hospital, the Affiliated Hospital of Nanjing University Medical School.
